# The effect of conversation on altruism: A comparative study with different media and generations

**DOI:** 10.1371/journal.pone.0301769

**Published:** 2024-06-14

**Authors:** Elie Maalouly, Ryuji Yamazaki, Shuichi Nishio, Marco Nørskov, Kohei Kamaga, Shoji Komai, Kiyoshi Chiba, Keiichiro Atsumi, Ken-Ichi Akao

**Affiliations:** 1 Graduate School of Engineering Science, Osaka University, Osaka, Japan; 2 Institute for Open and Transdisciplinary Research Initiatives, Osaka University, Osaka, Japan; 3 Department for Philosophy and the History of Ideas, Aarhus University, Aarhus, Denmark; 4 Faculty of Economics, Sophia University, Tokyo, Japan; 5 Faculty of Engineering, International Professional University of Technology in Tokyo, Tokyo, Japan; 6 School of Social Sciences, Waseda University, Tokyo, Japan; University of KwaZulu-Natal College of Health Sciences, SOUTH AFRICA

## Abstract

Despite the overwhelming evidence of climate change and its effects on future generations, most individuals are still hesitant to make environmental changes that would especially benefit future generations. In this study, we investigate whether dialogue can influence people’s altruistic behavior toward future generations of humans, and how it may be affected by participant age and the appearance of the conversation partner. We used a human, an android robot called Telenoid, and a speaker as representatives of future generations. Participants were split among an old age group and a young age group and were randomly assigned to converse with one of the aforementioned representatives. We asked the participants to play a round of the Dictator Game with the representative they were assigned, followed by an interactive conversation and another round of the Dictator Game in order to gauge their level of altruism. The results show that, on average, participants gave more money after having an interactive conversation, and that older adults tend to give more money than young adults. There were no significant differences between the three representatives. The results show that empathy might have been the most important factor in the increase in altruistic behavior for all participants.

## Introduction

Climate change has grown more noticeable in recent years and has had unique effects on climates all across the world. Climate change is predicted to have a wide range of effects, such as modifications to ecosystems [1], and impacts on human systems including water resources [2], forced human migration [3], and significant ocean acidification [4].

A lot of people are still hesitant to change their energy-related decisions and behaviors, despite the wealth of climate change evidence that is currently available. Many times, attempts to encourage people to adopt environmentally friendly practices have been somewhat unsuccessful [5, 6]. But why are we so apathetic and inactive if there is such a threat to future human generations? Do we lack compassion for future generations of humans? If so, how might we shape people’s altruistic behavior toward future generations? The preservation of our ecosystem and species may therefore depend on our ability to influence this altruistic conduct, as carelessness and negligence could have disastrous effects.

There are many other advantages to altruistic action besides merely environmental protection, including advantages to one’s health. Altruism is good for one’s emotional well-being and can significantly increase peace of mind [7], as well as enhance self-assurance, self-esteem, self-awareness, daily functioning, and lower depression [8].

Our goal is to find out if humans can be altruistic toward future generations of people and what factors may influence this behavior. Our main research question is the following:

*Does dialogue influence altruistic behavior toward future generations*?

And our sub-question is the following:

*If conversation is an influencing factor, are there differences in its effects based on age, appearance (looks of conversation partner), and personality*?

## Related work

### Altruism

Researchers have strong disagreements over what altruism is and how prosocial behavior is related to it. In an analysis by Pfattheicher et al. [9], in more than 74% of the articles published related to prosocial behavior or altruism, there was no relevant definition included. Therefore, it is crucial to define these concepts accurately in order to convey how we see altruistic behavior in our study.

Nearly all definitions have the commonality that prosocial and altruistic acts signify a “positive” behavior toward one or more persons. These actions are typically characterized as being done for the benefit of others, with the primary aim of promoting their well-being. The disagreement comes mainly in defining what behaviors are considered prosocial or altruistic. In this study, We shall approach prosocial behavior from an intentionalist perspective. This perspective emphasizes the intentional nature of the behavior. According to Batson et al., “prosocial behavior covers the broad range of actions **intended** to benefit one or more people other than oneself” [10].

From an intentionalist perspective, altruism can be defined as a subtype of prosocial behavior or as a motivational condition. For example, Batson defines altruism as “motivation with the ultimate goal of increasing another’s welfare” [11]. Similarly, Eisenberg defines altruism as “prosocial behavior which is not performed with the expectation of receiving external rewards or avoiding externally produced aversive stimuli or punishments” [12]. Therefore, prosocial behavior and altruism are two distinct but linked concepts in the intentionalist approach. Altruism refers to the purpose behind the action, whereas prosocial behavior solely involves the action itself.

We will use Batson’s definition of altruism moving forward, which states that it is “motivation with the ultimate goal of increasing another’s welfare”.

### Altruism in experiments

Altruism experiments provide considerable research obstacles. The basic goal of experiments on altruism is to eliminate any potential ulterior motive rooted in selfishness [13]. Because it provides a straightforward and comparatively pure evaluation of altruistic vs. self-interested behavior and is sometimes referred to as a measure of unconditional goodness [14], the Dictator Game [15] is commonly employed as a gauge of altruism. In the Dictator Game, a proposer decides a split to be divided between himself/herself and a respondent in which the proposer is free to choose any split they like and the respondent has no input in how the split is carried out [15]. The best (money maximizing) move in the Dictator Game is to keep the entire endowment, even though a sizable number of players choose to give money away. A meta-analysis of 131 studies by Engel found that dictators shared on average 28.35% of their endowment, with bimodal underlying distributions with peaks at 0% and 50% [16].

Even though the Dictator Game is a fairly popular way for researchers to gauge how benevolent people are, it is far from a perfect measure as there may be other potential motivations at play, such as demand characteristics [17] and self-signaling [18].

### Empathy and altruism

As in a study by Herne et al. [19], we differentiate between two types of empathy: an innate trait (permanent) and an induced state (temporary). Empathy has been measured as an individual trait or by generating an empathic state and assessing the connections of the trait or state empathy to altruistic conduct in order to explore the relationship between empathy and prosocial behavior [20].

Starting with trait empathy, one can divide it into two main categories: cognitive and affective. Cognitive empathy is the capacity to identify and comprehend another person’s mental state, whereas affective empathy is the capacity to share another person’s sentiments without experiencing any direct emotional stimulation oneself [21]. In a study by Jordan et al. [22] affective empathy appeared to be positively correlated to giving in a public goods game, but appeared to be negatively correlated to charitable donations. According to Edele et al. [23], allocations to a dictator game recipient and affective empathy are positively correlated, while there was no statistically significant correlation found between dictator game offering and cognitive empathy. Overall, it appeared that affective empathy is related to dictator game allocations in one study, whereas cognitive empathy does not appear to be a reliable predictor of dictator game allocations.

Let us next define empathy as an induced state. As we will be working within Batson’s definition of prosocial behavior and altruism, it’s critical to recognize the root causes of altruistic behavior. According to Batson, the major driver of altruistic behavior is the empathy-altruism hypothesis [24], where empathy refers to empathic concern. The empathy-altruism hypothesis states that empathic concern generates altruistic drive [25, 26]. Empathic concern is defined as the “other-oriented emotional response elicited by and congruent with the perceived welfare of a person in need” [11]. It generally entails feeling **for** the other instead of feeling **as** the other. In this study, induced empathy is defined as empathic concern.

The idea that empathy motivates altruistic behavior is supported by a wealth of research. The first supporting evidence was offered by Aronfreed [27] and Aderman [28], who each created experimental settings meant to encourage or discourage empathy for a person in need. Both researchers concluded that greater empathy was linked to more helping after finding that helping rose in the experimental conditions designed to foster empathy. Numerous trials [29–32] corroborated these conclusions. For a thorough review of the tests conducted to support this idea, we refer the reader to a review by Batson [26]. The empathy-altruism theory has been tested in over 30 tests, and the results to date have been positive.

The empathy-altruism hypothesis claims that altruistic motivation is produced by empathy, and this seems to be well supported by experimental evidence. But what induces empathy in the first place? In general, it appears that the two requirements that must be met in order to experience empathy in daily life are seeing the other as in need and valuing the other’s wellbeing [26].

In order to be able to perceive need one must be able to discern between another person’s current position and their ideal condition on one or more well-being dimensions [33–35]. These dimensions of well-being include the presence of positive affect, physical pleasure, security, and contentment, along with the absence of negative affect, bodily discomfort, anxiety, danger, stress, and sickness. The idea that empathy involves the feeling of need has been supported by evidence. From the study by Berger, participants were asked to watch a target person complete a task. He tricked the participants into believing that the target either received electric shocks (the electric shock condition) or did not (the no shock condition) [36]. The target also moved or did not move his arm in reaction to shock (a movement criterion). Everyone who participated in the trial was made aware that there would be no shocks administered during the experiment. Berger first claimed that both a painful stimulus (shock) and a distress reaction (movement) were necessary for an observer to infer that the target was experiencing pain (i.e., need). Second, he argued that in order for participants in his experiment to experience a physiological reaction when they deduced that the target was in pain—as opposed to fear or dread—or anticipation of the shock itself, they needed to be experiencing empathy for the target. Because only those who engaged in the shock/movement condition would come to this conclusion, Berger anticipated that only they would experience enhanced physiological arousal. In each of the other three scenarios, there was some participant information that was missing that would have allowed one to infer suffering. The outcomes met expectations. The notion that people can experience empathy when they observe another who is considered to be in need is supported by the fact that participants in the shock/movement condition were more physiologically stimulated when they saw the target than participants in the other three conditions. Subsequent research provided additional evidence to support this view [37, 38]. Despite the fact that the studies just mentioned demonstrate that people physically react when they perceive another person to be in need, the exploratory study by Stotland demonstrated through a series of experiments that this physiological reaction is a sign of empathy for others and that empathy can be boosted through perspective-taking [39].

Being aware of another person’s needs is insufficient. As was already established, appreciating the welfare of the other is necessary for the second prerequisite for experiencing empathy. If we don’t value the welfare of the person we perceive to be in need, we are less likely to take into account how they are impacted by a need. It’s usual to give someone’s welfare a low priority if we don’t like them. In that situation, realizing their plight can result in enjoyment at seeing their pain rather than sentiments of empathy [40–42]. Instead, placing a high value on someone else’s welfare makes it far more likely to adopt an other-oriented value appraisal of these circumstances and consider how this person is impacted by the events that occur in his or her life [26]. People typically have an innate tendency to place a positive value (or at least a moderate value) on the welfare of others, even complete strangers, as long as there are no evident reasons for antipathy [26].

In the context of economic games, Klimechi et al. [43] has shown Dictator Game giving being positively affected by inducing empathy. Powell et al. [44] showed that when affective empathy was strong and trust was low, inducing empathy increased Dictator Game giving. Nevertheless, other studies have shown no significant effects on Dictator Game giving with reported increases in empathy [19, 45].

### Dialogue and empathy

We can now investigate how conversation might impact empathy. Even though there hasn’t been much research that has examined the connection between conversation and empathy, there have been some significant findings that seem to support it. According to Nishida, verbal and nonverbal communication are both used during conversations [46]. Joint actions and words between two people, such as engaging in similar behaviors and using comparable language, improve communication in dialogue. The study by Gould et al. examined the link between empathy and conversational pleasure [47]. Their research found a connection between older adults’ reported levels of happiness with their interactions and their degree of empathy for others. The subject, duration, and enjoyment of the conversation can vary depending on who is speaking. Little study has been done on the kinds of interactions that can foster empathy, despite evidence linking empathic concern and conversational satisfaction [47]. On the other hand, self-disclosure has been widely used as a concept to assess the rise in intimacy [48]. According to Morton, disclosure can be viewed as either factual and descriptive or emotive and judgmental [49]. Greater emotions are involved in emotional disclosure than in factual disclosure, which lacks greater connection and is more impersonal. The degree of intimacy felt between individuals and emotional disclosure may be related [50]. Even while disclosure is not necessary in every conversation, these results suggest a connection between the type of disclosure used and improved empathy. Finally, Andreoni has shown that, compared to one-way communication and no communication at all, two-way verbal communication between players of a Dictator Game evoked a higher level of altruistic behavior from allocators [51].

Other findings that support the connection between conversation and empathy are in the context of democratic deliberations. Muradova et al. [52] argues that when given the correct circumstances, deliberation encourages individuals to actively imagine the experiences, viewpoints, and sentiments of others, a process known as perspective-taking, which results in more contemplation when citizens make decisions. In a study on citizen deliberation on immigration, the findings imply that individuals with restrictive initial views regarding immigration were particularly impacted by participating in discussions in groups with differing viewpoints; they became far more open to comprehending the ideas of immigrants. Additionally, in the like-minded treatment, discussion resulted in a rise in out-group empathy [53]. A study by Herne et al. [54] looked at how communication and deliberation affected players’ choices on investment in a common-pool resource game where choices were made in an asymmetrical and predefined order. According to their findings, simple communication was sufficient to address issues with common-pool resource usage, but deliberation also helped to reduce power disparities when it came to investment choices.

### Personality

The five-factor model of personality has been used in numerous studies to link personality traits to Dictator Game altruism. However, the results were inconsistent. In the Dictator Game, none of the conventional five characteristics accurately predicted altruism. Even agreeableness, which is typically thought to be one of the five traits that should positively predict altruistic behavior, has only been shown to positively predict Dictator Game altruism in some studies [55–57] but not in others [58–60].

Another model of personality in common use is the HEXACO model, which extends the conventional five-factor approach to include a sixth fundamental personality element called Honesty-Humility (HH) [61]. This element simply refers to “the tendency to be fair and honest in interacting with others, in the sense of working with others even when one may exploit them without incurring retaliation.” Fairness, greed avoidance, sincerity, and modesty are all included in this element. As a result, HH incorporates a number of agreeableness traits, particularly those related to nonexploitation. Accordingly, HH has been associated with Dictator Game giving in several studies [62–64] that relied on fictitious incentives, as well as one study that used an incentivized Dictator Game with real allocations [65]. Compared to the remaining personality characteristics of the HEXACO model and the five-factor model, HH was found to be a better predictor of Dictator Game giving [65]. In a meta-analysis by Thielmann et al., HH appears to be the best predictor of Dictator Game giving among all the remaining factors of the five-factor model and the HEXACO model [66].

### Age

Is there a general tendency for prosociality and altruism to rise with age over the course of a person’s lifetime? There seems to be emerging evidence that older adults exhibit more prosocial and altruistic behavior than young adults [67] across a multitude of measures such as economic games, learning for others, effortful actions, and charitable donations. In a study on 408 residents from Tokyo, Japan, prosocial behavior appeared to increase with respect to age across five economic games [68]. In a meta-analysis combining 16 studies evaluating altruism in 1,581 younger and older adults using measures of altruism ranging from economic games to self-reported measures, an age-related difference in altruism was found, with older persons exhibiting more altruism than younger adults [69].

When incentives were won for someone else instead of themselves, older adults learned more efficiently than younger adults, according to a recent study by Cutler that employed computational reinforcement learning models [70]. Older adults were also shown to be willing to exert more effort to benefit others when compared to younger adults [71].

Several studies have shown that older adults may be more willing to donate to charities compared to younger people [72–75]. The majority of studies, however, only included individuals from Western, wealthy, educated, industrialized, and democratic nations. In a study by Cutler with 46,576 participants in 67 countries, age was a good predictor for prosocial behavior, where older adults were more willing to donate to hypothetical charities compared to younger people [76].

### Physical appearance

One of the most famous examples of the consequences of the physical appearance of a robot is the uncanny valley theory [77]. According to the uncanny valley theory, a robot’s likeability and its resemblance to a human are positively correlated; However, there is an abrupt decline in likeability at very high degrees of human resemblance. Another effect of the physical appearance of a robot is the potentiality for anthropomorphization: the propensity to assign human attributes to non-human agents, such as animals or computers [78]. An increase in potential anthropomorphization occurs as a result of a robot’s physical resemblance to humans. The impact of anthropomorphization on altruistic intention has been studied by Riek et al. [79]. A movie clip depicting one of five protagonists, which includes a human boy and four robots with physical attributes ranging from those of a Roomba to a human-like android, was shown to participants. Film clips were either neutral or showed the protagonist being treated horribly and cruelly. “Which of the four robots would you save in the event of an earthquake?” was the question that the subjects were asked after watching the video. Participants felt more empathy toward the human-like robots and reported they would take greater risks in order to save them.

In addition to physical appearance, robots with human-like qualities are perceived more like humans. Numerous research on human-robot interaction demonstrates that giving robots a “humane” quality (such as thinking, planning, or showing melancholy) might cause them to be perceived more like humans, and therefore advantageous for interaction by successfully encouraging prosocial conduct [80] and teamwork [81, 82]. Much evidence seems to support this strategy, showing the rise in prosocial attitudes toward social robots [80, 83–85] and improvements in human-robot collaboration [86–89]. Chi et al. [90] have demonstrated that occasionally, people’s faith in robots with human-like traits may increase.

Studies involving both human and non-human participants in experimental economic games are very limited. A meta analysis by Johnson and Mislin [91] showed that playing trust games with a real person resulted in a bigger shared endowment compared to playing against a computer. These results, appear to be at odds with past research, which has more consistently demonstrated that rejection rates are significantly lower when offers are made by a machine as opposed to a human player [92, 93]. In a study by De Kleijn et al. [94] where human participants were involved in a Dictator Game and Ultimatum Game with one of four possible recipients (human, humanoid, hexapod, and laptop), they found an influence of appearance on Dictator Game results but not on Ultimatum Game results. However, their results were difficult to properly interpret. Additionally, in such games with robots as recipients, giving money to a robot might seem illogical to some participants and might make the interpretation of the results difficult especially when compared to a human recipient.

### Approach and hypothesis

Due to dialogue’s capacity to induce empathy, we think it has a strong potential to encourage altruistic behavior. Therefore, we think conversation may be a useful method for fostering altruistic behavior since we are operating within Batson’s definition of altruism and prosocial behavior as well as within the empathy-altruism hypothesis, which states that empathy is a fundamental source of altruistic conduct. Through conversation, it is possible to recognize the other as in need and give their welfare a high priority, which are the two conditions that must be met to encourage empathy in people according to the empathy-altruism hypothesis. We think that inducing empathy will motivate people to take action with the goal of benefiting the other person. In addition to the hypothesized effect of conversation on all participants, we believe that the age of the participants will also have an impact on altruistic behavior, where older adults might show more altruistic intention than young adults. The physical appearance of a conversation partner might have an effect, even though the research comparing humans and machines in how they affect altruistic behavior is still small and inconclusive. And finally, affective empathy and personality of the participants, and specifically the Honesty-Humility dimension, might have an influence on the participants’ altruistic behavior.

Our potential contributions to this paper are as follows:

- Showing that conversation can be effective in fostering altruistic behavior.
- Showing that the effect of conversation on altruistic behavior varies depending on the age of participants.
- Showing that the effect of conversation on altruistic behavior does not change with respect to the physical appearance of the conversational partner.

Participants in this study were randomly assigned to one of three experimental conditions: the “human condition”, the “robot condition”, or the “speaker condition”. Each experimental condition matches the conversation partner that participants interacted and engaged with in dialogue. The conversation partner will be henceforth referred to as “medium”. In the “human condition” the participants were introduced to a human and told that the medium is an actor role-playing as a human from the future. In the “robot condition” the participants were introduced to an android robot, and in the “speaker condition” they were shown a bluetooth speaker (connected to a hidden computer). In the “robot condition” and “speaker condition”, the participants were told that the medium is controlled by an AI living in a simulation of our world and is currently living in the future. The participants were asked to play a round of Dictator Game with the medium (round 1) where it was made clear that the medium represents a charity organization for environmental preservation. The participants then engaged in an interactive conversation with the medium about life in the future and how it has been affected by climate change before playing another round of Dictator Game (round 2) where again the medium was a representative of the same charity organizaiton. Our hypotheses are the following:

H1. The participants will report higher levels of empathy in round 2 compared to round 1 due to the conversation’s ability to induce empathy.
H2. The participants will share more money in round 2 compared to round 1, due to the induced empathy as per the empathy-altruism hypothesis.
H3. Older adults will share more money than young adults, as studies have shown that older adults exhibit more prosocial behavior than young adults.
H4. The medium in the “robot condition” will receive more money than the “human condition” and “speaker condition”. We believe that the information received from an AI living in the future might be more credible than a human actor which would give an advantage to the robot and speaker. Additionally, the human and robot might have an advantage over the speaker due to the effects of anthropomorphism. Therefore, we hypothesize that the robot will receive more money due to both of the aforementioned effects.
H5. The Honesty-Humility dimension of personality will positively moderate the effect of conversation on Dictator Game giving, as studies have shown that Honesty-Humility have been shown to positively predict Dictator Game giving.
H6. Affective empathy will positively moderate the effect of conversation on Dictator Game giving. Even though there has been limited evidence on the ability of affective empathy to predict Dictator Game giving, we believe the combined effect of affective empathy and the ability of conversation to induce empathy would positively affect Dictator Game giving.

## Materials and methods

### Experimental conditions

**Fig 1:**
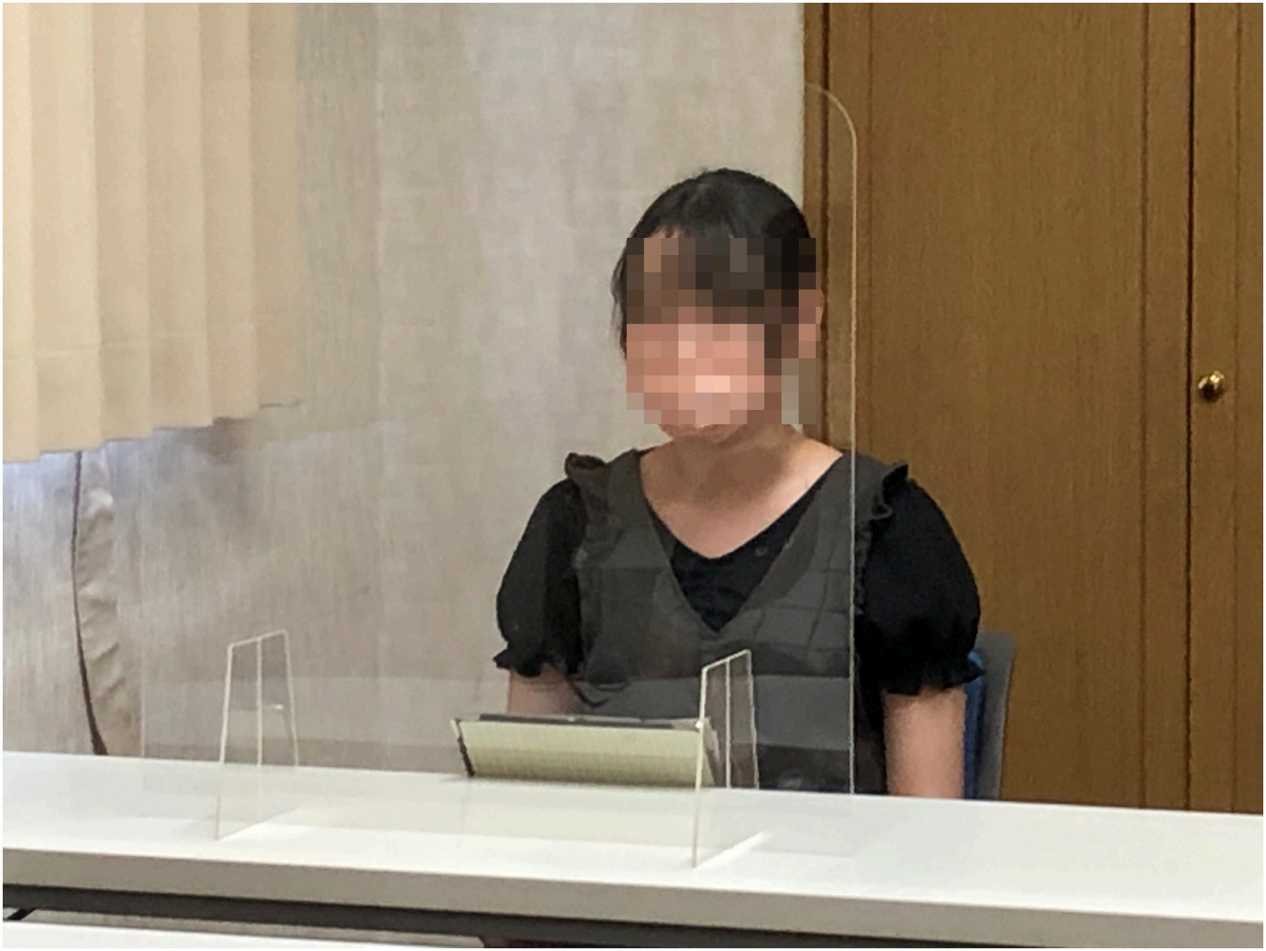
The human condition. The staff member who role-played as a human from the future in the “human condition” of the experiment.

**Fig 2:**
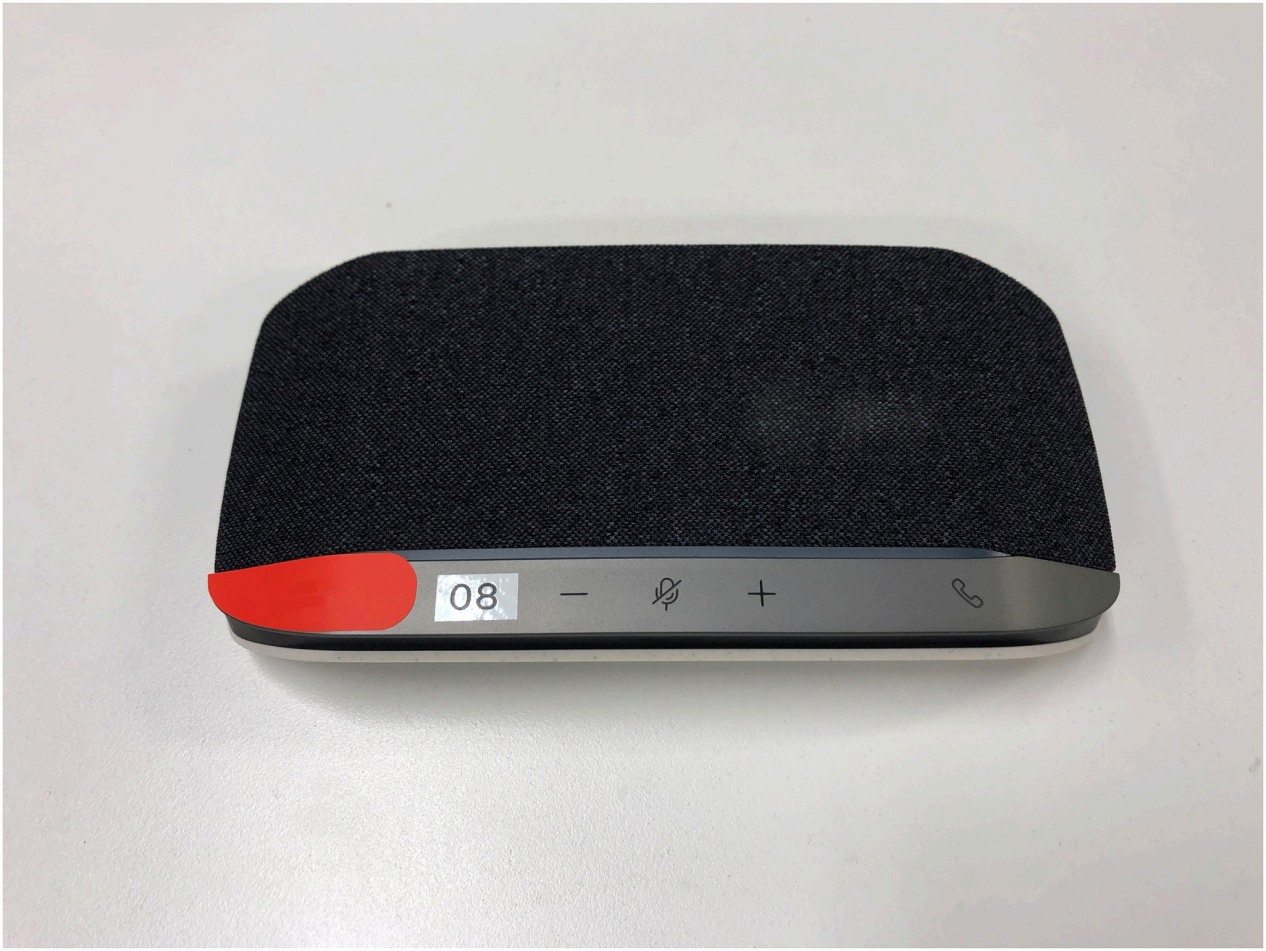
The speaker condition. The speaker used for the “speaker condition” of the experiment.

**Fig 3:**
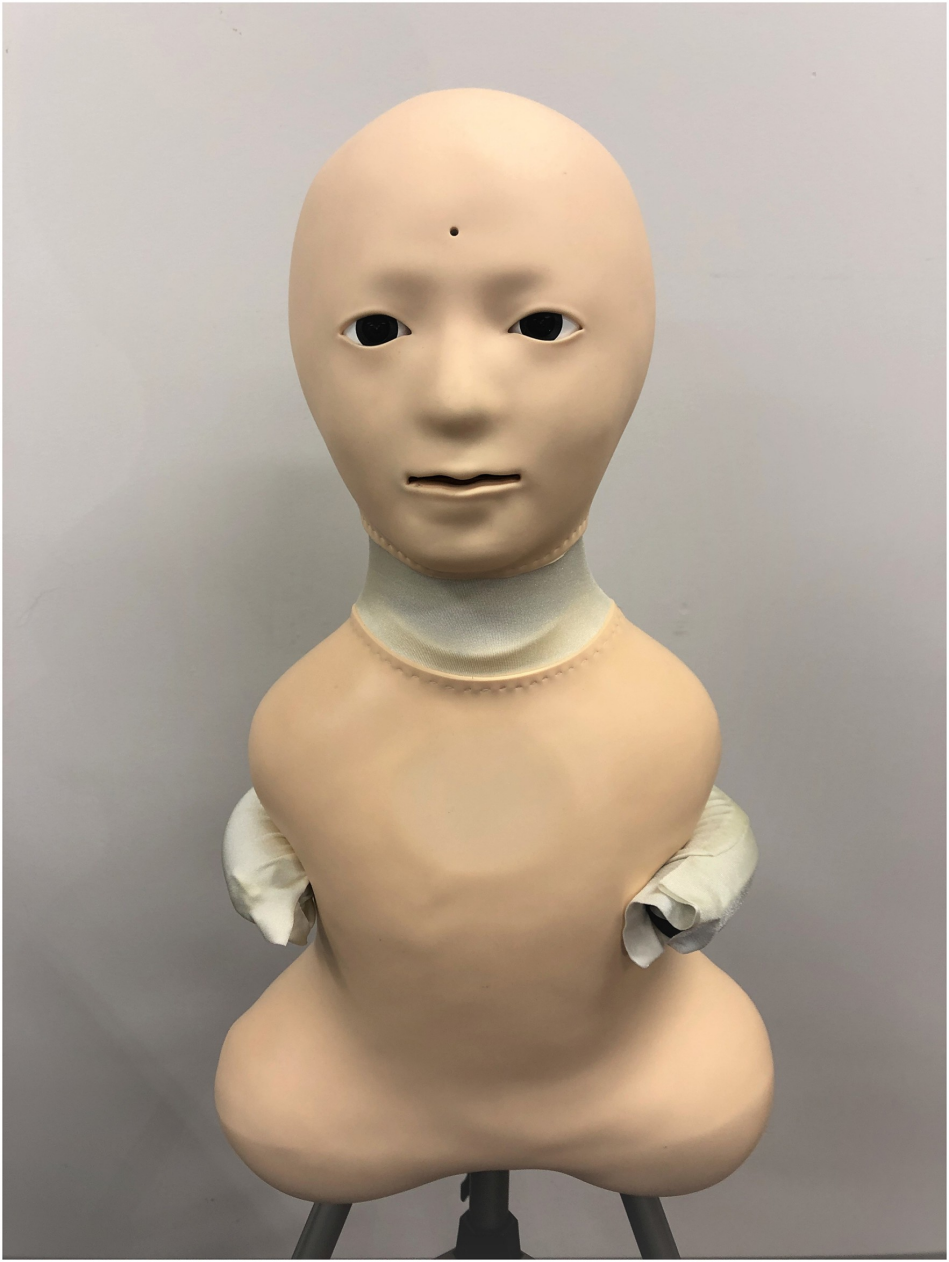
The robot condition. Telenoid, the robot used for the “robot condition” of the experiment.

**Fig 4:**
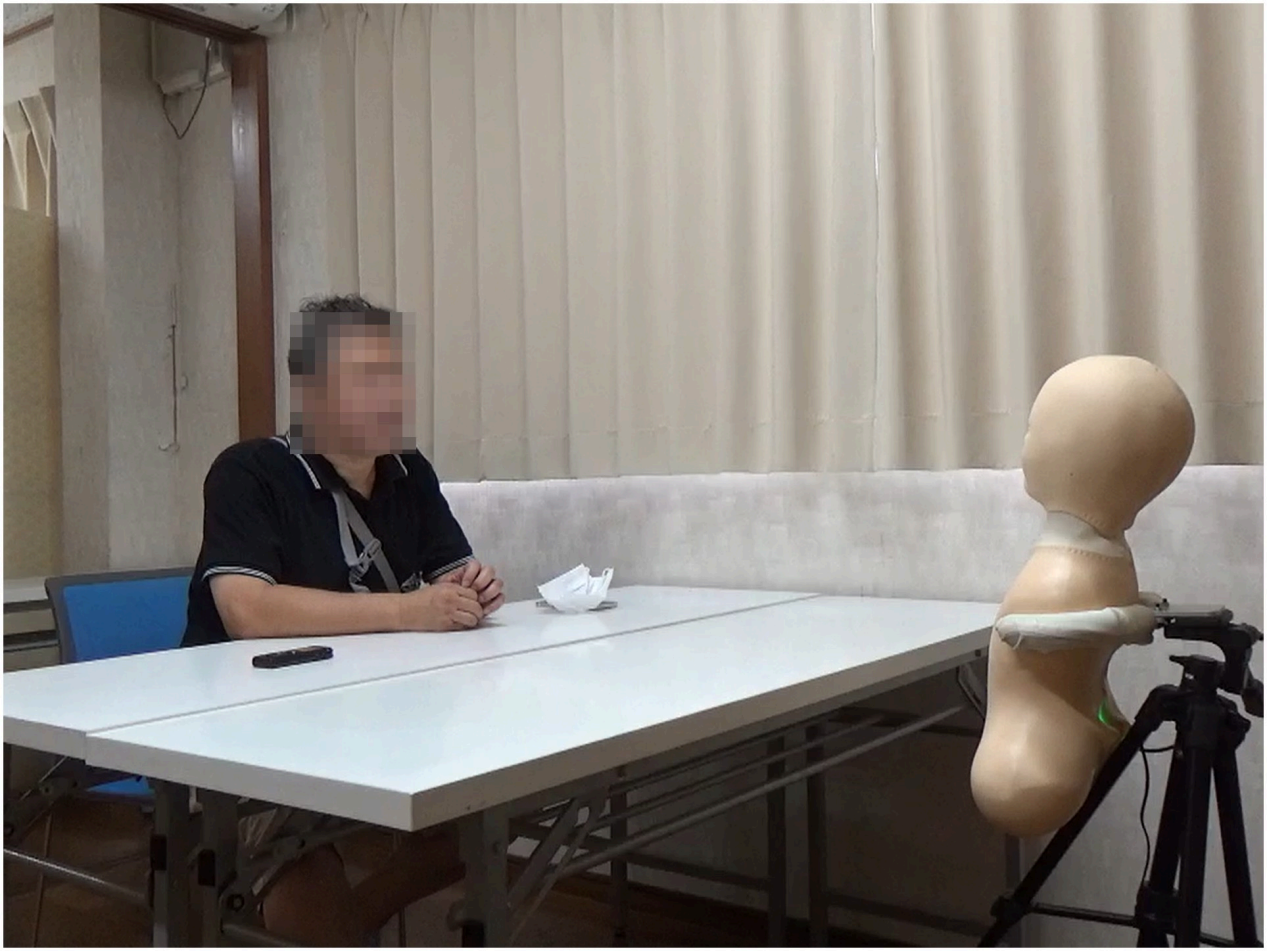
Experimental setting. The medium is seated across from the participant seperated by a table.

**Fig 5:**
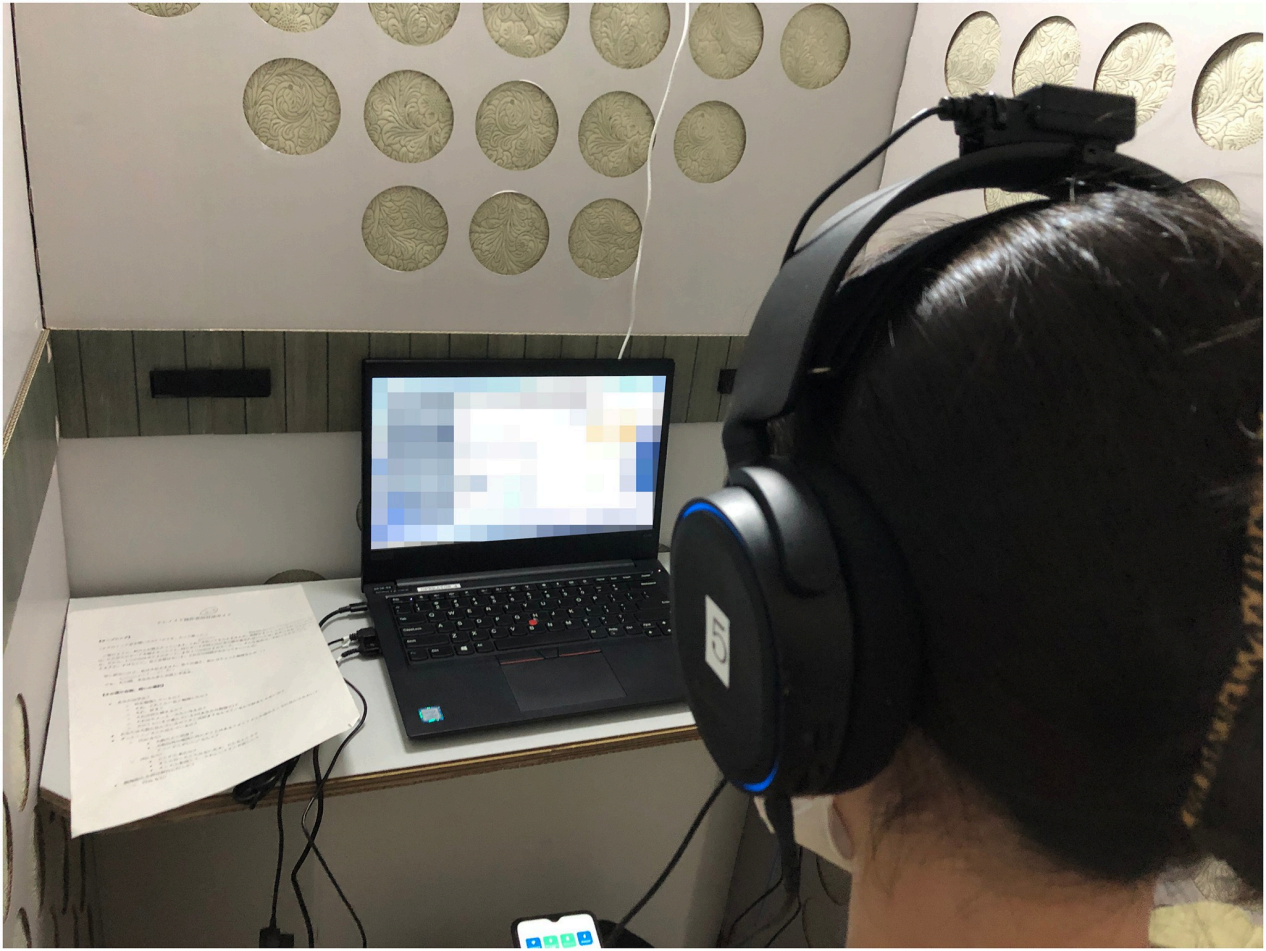
Robot teleoperation. A staff member teleoperated the robot from a separate room in the facility. The teleoperator can see the participant on the screen through the camera integrated into the robot, and can control the head movement of the robot through a motion sensor mounted on the headphones.

Three experimental conditions were set up for this study. In each of these conditions, a medium was used as the representative of future generations. A human for the “human condition” shown in Fig 1, a speaker for the “speaker condition” shown in Fig 2, and a robot for the “robot condition” shown in Fig 3. In the case of the robot condition, the participants were introduced to a robot that was placed across a table from them as shown in Fig 4. The robot was teleoperated by a staff member from another room as shown in Fig 5, but the participants were told that the robot is controlled by an AI living in a simulation of our world and is currently living in the year 2220. In the case of the speaker condition, the participants were shown a Bluetooth speaker on a table in front of them. The speaker was connected to a computer controlled by a staff member; the participants were told that the speaker was connected to a computer running an AI that is living in a simulation of our world and is currently living in the year 2220. Finally, for the human condition, the participants were introduced to a staff member who was seated across a table from them. They were told that this person is going to role play as a person from the future living in the year 2220.

We believe that the differences between these three conditions will ultimately lie in the appearance of the medium and the believability of the scenario. Regarding the believablity of the scenario, stating that the robot and speaker are both controlled by an AI might be believable for some people which we think would increase their trust in the medium, and thus the information they hear might seem more credible. We think this may give the speaker and robot an unfair advantage over the human, as no one would believe the human to be actually living in the future. The scenario we proposed for the human condition, which is stating that the human is an actor that will role-play as a person from the future would give the human condition a fairer condition. Regarding appearance, the human and the android are superior to the speaker in this regard. The robot might fare better than the speaker due to the possibility of embodied interaction which leads to more engagement in interaction [95]. The robot we used for the “robot condition” is Telenoid [96]. It was created with the goal of eliminating as many human traits as possible without sacrificing essential communicative abilities. The outcome is an android that can stand in for any person and has a neutralized appearance.

The Telenoid is a 50 cm, 3 kg teleoperated robot that allows a remote operator to control its head movement while speaking through it. In order to teleoperate the robot, it is necessary to have a laptop linked to the same network as the Telenoid along with headphones, a motion sensor, and a microphone. The Telenoid’s onboard camera and microphone provide a real-time video and audio feed to the operator, who is situated in a different, isolated room. A motion sensor mounted on top of the headphones allows the Teleoperator to control the Telenoid’s head movement. The Telenoid’s head motion will resemble the operator’s in this fashion.

### Procedure

Before the experiment began, the participants were given time to fill out a 60-item NEO-FFI personality test, a 100-item HEXACO-PI-R personality test, a 24-item MES empathy test, and an environmental awareness questionnaire.

After the participants finished filling out the questionnaire and all the tests, they were then taken by a staff member to a room and seated in front of a table. Across the table from where they sat, they were introduced to one of the three media (human, speaker, and robot) depending on their experimental condition. An explanation was given to them about the medium sitting in front of them, as explained in the previous section.

Before speaking to the medium, the participants were asked to play a round of the Dictator Game with the medium. After the participant decides on a split for the coins, they were asked to fill out a questionnaire to explain their choice in the game and their first impressions of their opponent. With this, the first round was done and the second round began with a 10 minute interactive conversation that took place between the participants and the medium. The conversation was highly controlled so it would be the same for all media.

**Fig 6:**
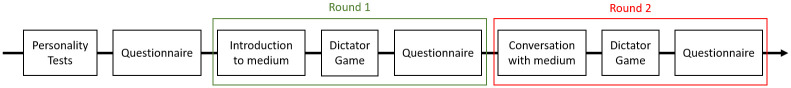
Experimental flow. The figure shows the flow of the experiment.

After the conversation, the participants were asked to play another round of the Dictator Game with their opponent with the same settings as the first round. The participants were later asked to fill out the final two questionnaires. One questionnaire was for understanding their choice in the game and their current impressions of the medium, and another questionnaire was to evaluate the credibility of the scenario (if and to what extent they believed the scenario presented of the future world). The full list of questions from all the questionnaires used in this study is included in S2 File. The experimental flow is shown in Fig 6. Our trial in this study was conducted in compliance with the Helsinki Declaration, and prior to the trial, we received written informed consent from all participants, based on approval for the trial from the Ethics Committee at the School of Engineering Science, Osaka University (approval code: R2-6-4). The individuals in Figs 1 and 4 have given written informed consent (as outlined in PLOS consent form) to publish their image alongside the manuscript.

### Personality and empathy tests

Two personality tests were used in this study. The NEO Five-Factor Inventory (NEO-FFI) [97] was used for assessing the personality of participants across five personality dimensions based on the five-factor model. Each of the five dimensions of Neuroticism, Extraversion, Openness, Agreeableness, and Conscientiousness has twelve items. On a 5-point Likert scale, each item is answered (strongly disagree, disagree, neutral, agree, strongly agree). In Japan’s general populace, the Japanese version has proven to be reliable and valid [65]. The second personality test we used was the 100-item version of the HEXACO-PI-R [98]. It was used to assess the personality of participants across the six personality dimensions: Honesty-Humility, Emotionality, Extraversion, Agreeableness, Conscientiousness, and Openness to Experience. The Japanese version has been proven to be valid across Japan’s general populace [99]. The final test performed by the participants was the Multidimensional Empathy Scale (MES) [100]. It is a 24-item self-report measure across two dimensions of cognitive empathy (perspective taking, and fantasy), and three dimensions of affective empathy (other-oriented emotional response, self-oriented emotional response, emotional susceptibility).

### Conversation

In order to have all the participants participate in the same conversation regardless of experimental condition, the conversation had to be highly controlled. But, we also wanted the participants to engage in an interactive conversation as opposed to a one-sided conversation where they would only listen to what the medium had to say without any sort of participation. To obtain a highly controlled interactive conversation, we developed a script that the medium would follow. The script consists of a set of pre-written phrases for the medium to say, after which the medium asks a question to the participant. After the participant’s reply, the medium responds with a generic response such as, “I see” or “that’s interesting”. And the conversation repeats with pre-written phrases from the medium.

The conversation revolves around life in the future, the positives and the negatives. Some of the positive things discussed by the medium include technological advances such as flying cars and efficient energy sources. The negatives discussed by the medium include rising temperatures, food shortage, and increased occurrence of natural disasters.

The full script used in this experiment is included in S1 File.

### Dictator Game

A total of 1000 Japanese Yen in 100 Yen coins were placed on the table in front of the participant. They were asked to split the coins with their opponent in any way they desire. It was made clear that they do not have to share any of the coins with their opponent, and that they are free to choose any split, should they choose to; however, the shared amount will be deducted from their reimbursement. At the end of the experiment, all the participants were fully reimbursed regardless of their splits in the game and they were asked to not discuss any of the details of this experiment with other participants.

The participants were told that their opponent in the game is a representative of a charity organization committed to environmental preservation. The reasoning behind this decision is that the results of the Dictator Game would normally be used to evaluate the participants’ behavior toward their opponents; however, in our case, we are more interested in participants’ behavior toward people in the future, and not to their opponent specifically. Therefore, we think it’s important that the Dictator Game results should generalize to people in the future as a beneficiary of the Dictator Game donation. Choosing to give money to a charity organization for environmental preservation would indirectly benefit the people living in the future. This would also solve another problem brought upon by the differences in the media of this experiment. Participants might be more willing to give money to a human compared to a machine as it would not be clear to whom the money is going (in the case of the robot or speaker). Participants might be highly hesitant to let go of part of their earnings to a machine that has no use for money. Another issue is that in our definition of altruism, there has to be a recipient benefiting from some behavior. In the case of a human, the recipient’s increase in wealth is an obvious benefit from the participants’ behavior in the game; However, in the case of a machine in the Dictator Game, this same behavior from the participants might not be regarded as beneficial to the machine. Therefore, concluding that the money shared with a machine in the Dictator Game is a sign of altruistic behavior would be highly questionable.

### Participants

Sixty participants participated in the experiment between the 20th of June and the 5th of July, 2022. All 60 participants successfully completed the experiment. Authors EM and RY had access to information that could identify individual participants during and after data collection. However, the information was separated from the results after data collection, and each participant’s data was assigned to a unique ID in order to perform data analysis and ensure the anonymity of the participants. The recruited participants were equally split between a young age group consisting of university students with a mean age of 21.63 and a standard deviation of 1.54, and an old age group with a mean age of 66.27 and a standard deviation of 2.61. The participants were equally split between males and females across age groups. The 30 participants in the young age group were recruited between the 2nd of May and the 5th of July by handing out fliers at Osaka University, and by posting an online advertisement. Participants in the old age group were recruited between the 22nd of April and the 1st of July by asking for participants from older adults registered at a silver human resource center, which is a program that provides part-time, paid employment to retirement-aged adults. All participants were reimbursed with 3,000 Japanese Yen upon completing the experiment.

Twenty participants were assigned to one of the three experimental conditions, such that ten participants consist of the young age group and ten consist of the old age group. And the ten participants in each age group consist of five male and five female participants.

## Results

### Conversation

The alpha level was set at *p* = 0.05 for all the statistical analyses.

To evaluate the effect of conversation on empathy and altruistic behavior (**H1** and **H2**), the Dictator Game and the result of questionnaire item 4 of round 1 and round 2 were compared. The Dictator Game results were compared using parametric tests (paired t-test) as the results were not skewed enough to violate the normality distribution assumption (as visualized on a QQ plot). The tests are two-tailed and report a p-value, and the effect size (Cohen’s d). For all questionnaire items, we utilized non-parametric tests (Wilcoxon signed-rank test) for consistency, because some of the questionnaire data exhibited some degree of skewness and revealed a violation of the normality distribution assumption. The tests are two-tailed and report the p-value, 95% confidence intervals, and the effect size (r).

From item 4 in the questionnaire (Q4: Did you feel empathy for the medium?), the median score has significantly increased from round 1 to round 2 (round 1: *Mdn* = 0, *IQR* = 2; round 2: *Mdn* = 1, *IQR* = 2. Wilcoxon signed-rank test, *p* < 0.0001, *r* = 0.57). The conversation seems to have been effective at inducing empathic feelings from the participants towards the medium. This result confirms **H1** that participants will report feeling more empathy towards their opponent in round 2 compared to round 1.

**Fig 7:**
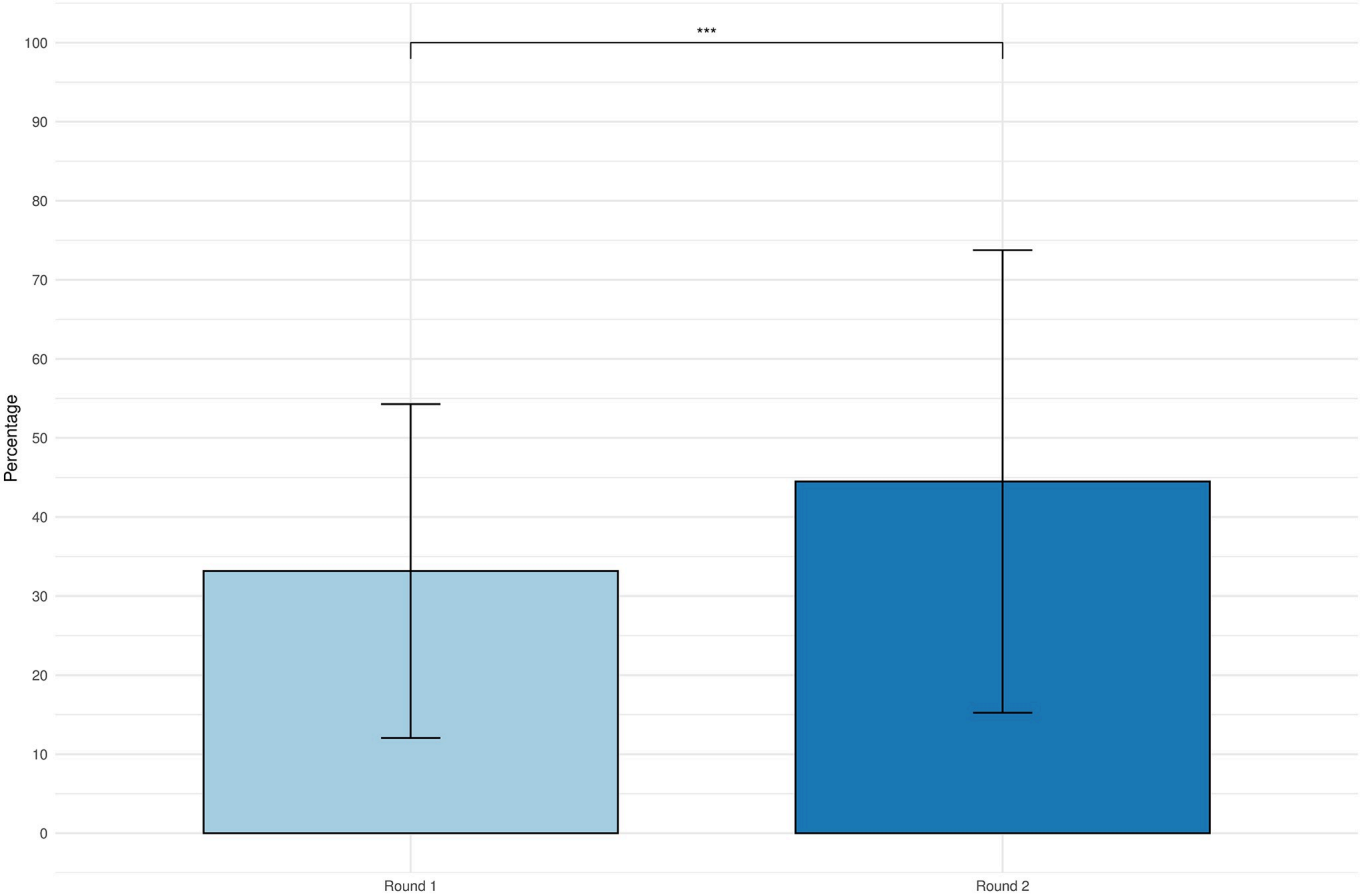
Dictator Game money distribution. The bar plot shows the average distribution of the percentage of money shared with the media in each round of the experiment. The error bars represent the standard deviation. The vertical axis represents the percentage of the total amount of money shared. Round 1 is the first game played by the participant after being introduced to the medium. Round 2 is the second game played by the participant after having an interactive conversation with the medium.

The average percentage of money shared with the media was significantly higher after the conversation (Round 1: 33.2 ± 21.1; Round 2: 44.5 ± 29.3. Paired t-test, *t*(59) = −4.04, *p* = 0.0002, *d* = −0.521). The Dictator Game results are shown in Fig 7. From the figure, it is apparent that the amount shared in the Dictator Game has risen on average from 33% to 45%. The result confirms **H2** that participants will share more money with their opponent in round 2 compared to round 1.

### Age and experimental condition

To evaluate the differing effects of conversation across age groups and media types (**H3** and **H4**), a three-way mixed ANOVA was performed on the Dictator Game results where “conversation” (round 1 and round 2) is the within-subject factor, and “age group” and “experimental condition” were the between-subject factors.

The data was checked for normality using Shapiro-Wilk’s test of normality. Out of the twelve cells, two cells failed the normality test. Using a QQ plot, the points in all the cells fell approximately along the reference line. Therefore we can assume normality of the data. There was homogeneity of variances as assessed by Levene’s test of homogeneity of variances.

The three-way ANOVA showed a statistically significant three-way interaction between conversation, age, and experimental condition (*F*(2, 54) = 5.75, *p* = 0.005). Additionally, there was a main effect of age (*F*(2, 24) = 14.23, *p* < 0.001).

Simple simple pairwise comparisons were run between the different age groups before and after the conversation. A Bonferroni adjustment was applied. The average amount given in the Dictator Game was statistically significantly different between the young age group and the old age group in round 1 (*p* = 0.003) and in round 2 (*p* = 0.001).

**Fig 8:**
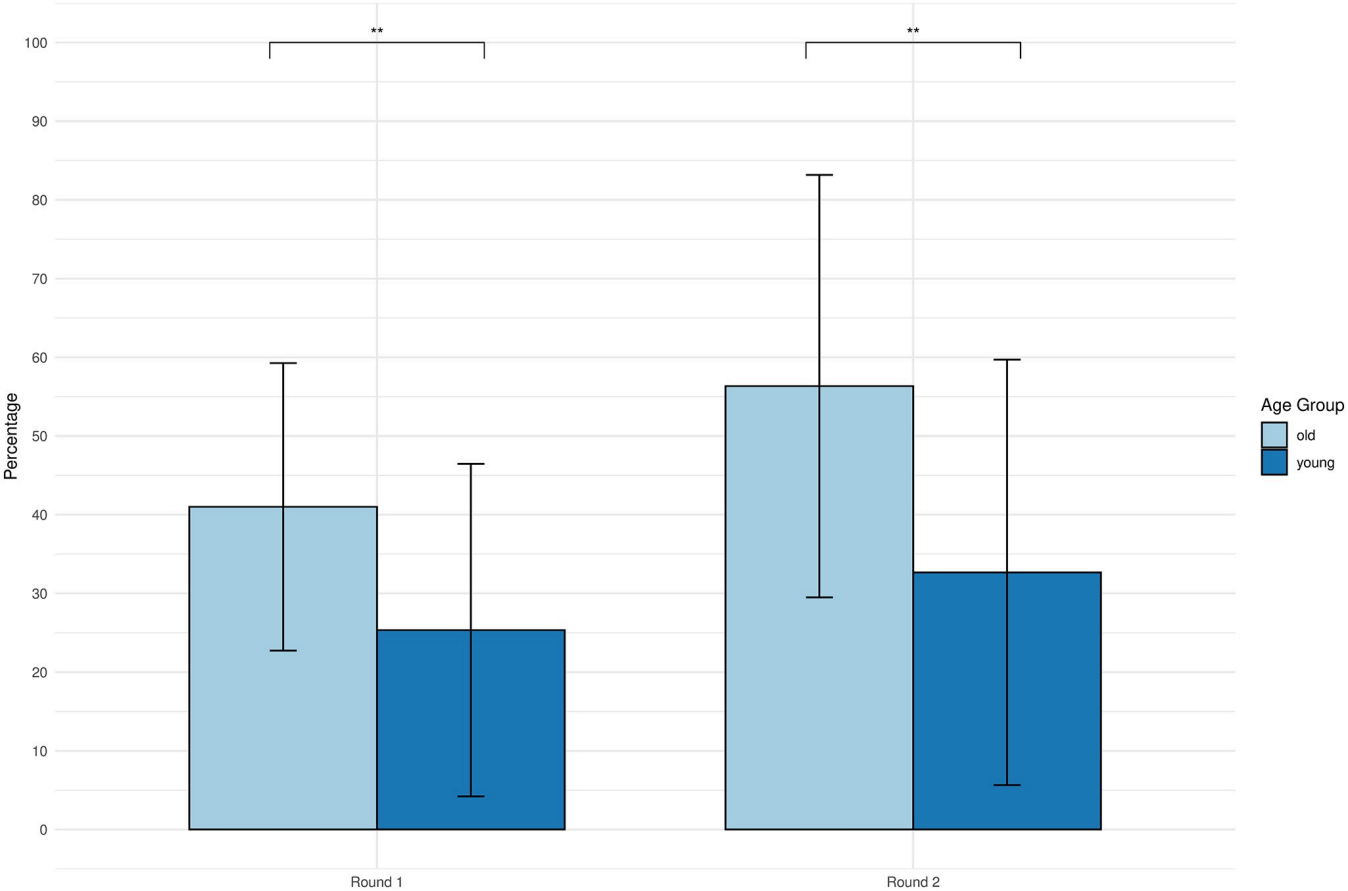
Dictator Game money distribution across age groups. Dictator Game money distribution between the old age group and the young age group in each round. The vertical axis represents the percentage of the total amount of money shared.

The results are shown in Fig 8. In round 1, young people, on average, shared 25% of their endowment, while older adults, on average, shared 41%. In round 2, young people, on average, shared 33% of their endowment, while older adults, on average, shared 56%. The result confirms **H3** that on average older adults tend to give more money in the Dictator Game compared to younger people.

For the simple two-way interactions and simple simple main effects, a Bonferroni adjustment was applied. There was a statistically significant simple two-way interaction between age and experimental condition after the conversation (Round 2) (*F*(2, 54) = 6.05, *p* = 0.004), but not before the conversation (Round 1) (*F*(2, 54) = 0.52, *p* = 0.60).

There was a statistically significant simple simple main effect of experimental condition on the Dictator Game results for the old age group after the conversation (*F*(2, 27) = 4.03, *p* = 0.029), but not for the young age group (*F*(2, 27) = 2.44, *p* = 0.11).

All simple simple pairwise comparisons were run between the different conditions for the old age group after the conversation. A Bonferroni adjustment was applied. The average amount given in the Dictator Game was statistically significantly different between the human and the speaker (*p* = 0.025). There was no significant difference between the human and the robot (*p* = 0.54) and between the robot and the speaker (*p* = 0.46). The results therefore do not confirm **H4** that the robot would receive more money from the participants compared to the other media.

**Fig 9:**
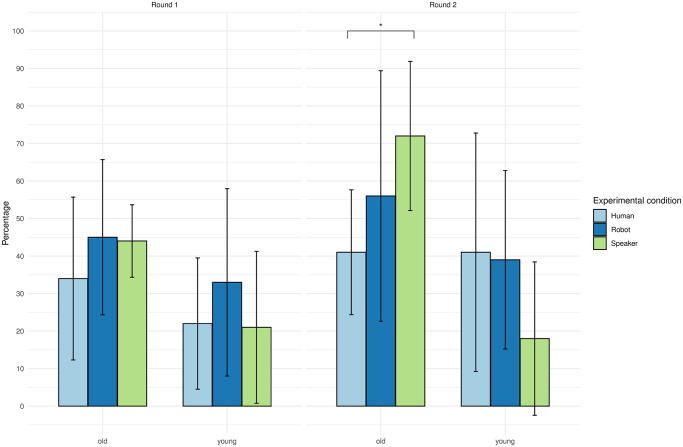
Dictator Game money distribution across age groups and media types. Dictator Game money distribution along the factors of time (round 1 and round 2), age groups (old age group and young age group), and media (human, robot, and speaker).

The results of the three-way mixed ANOVA are shown in Fig 9.

### Personality and trait empathy

Regression analysis was used to evaluate the effect of personality and trait empathy on Dictator Game results (**H5** and **H6**). We performed a moderation analysis to test whether personality or trait empathy moderates the effect of conversation on Dictator Game giving in a linear model. The dependent variable is the percentage shared in the Dictator Game, and the Personality / empathy traits are the independent variables. Time is used as a dummy variable that can take on two values, 0 for round 1 (pre-conversation) and 1 for round 2 (post-conversation). And finally, the participants are added as a random effects variable. The model is shown in Eq (1) where DG represents the percentage of money shared with the medium in the Dictator Game, F is one of the personality / empathy factors, T is a dummy variable for pre and post-conversation and Se is a by-participant random intercept.


$$\text{DG}=b_{0}+b_{1}F+b_{2}T+b_{3}FT+Se$$ (1)

None of the dimensions of personality in the NEO-FFI and HEXACO-PI-R showed a significant interaction with *time*. The results do not support **H5** that the Honesty-Humility dimension of personality will be able to positively moderate the effect of conversation on Dictator Game giving.

The moderation analysis on the factors of the MES test showed a significant interaction between *affective empathy* and *time*. The interaction estimate was 1.86 with a p-value of 0.003. The 95% confidence interval for the interaction effect is (0.68 to 3.05). We therefore reject the null hypothesis and conclude that there is evidence that the affective empathy by time interaction effect is not 0, *t*(58) = 3.14, *p* = 0.003.

In conclusion, we only found a significant *affective empathy* by *time* interaction effect. The difference in Dictator Game giving increase rate is 1.86 percent per point score on the *affective empathy* scale, with a 95% confidence interval from 0.68 to 3.05. This result confirms **H6** that affective empathy will positively moderate the effect of conversation on Dictator Game giving.

In S3 File, we report the complete results of the regression analysis of all the personality and empathy tests.

## Discussion

### Effect of conversation on altruistic behavior

We hypothesized that the dialogue would increase altruistic behavior as measured by Dictator Game donations. The results show a statistically significant increase in Dictator Game giving between round 1 and round 2 of the experiment, indicating there was a significant influence from the conversation on participants. Participants, on average, increased their donations from 33% in round 1 to 45% in round 2 which appears to be in agreement with our previous study [101]. Hence, it is clear that participants’ attitudes regarding the media have changed. The participants’ participation in a dialogue with the media distinguished the two rounds. These findings demonstrate a strong effect of two-way verbal communication on altruistic behavior as indicated by the Dictator Game.

From item 6 of the questionnaire, it is apparent that the conversation was successful in inducing empathy toward the medium. Additionally, from the MES empathy test that was administered, the affective empathy appears to have a significant interaction with the effect of conversation on the amount shared in the Dictator Game. In other words, the increase in the amount shared in the dictator game was higher for participants with a higher affective empathy.

Therefore, from our results, empathy might seem to be the driver of altruistic behavior. However, its effect might be limited. Through the conversation, the participants might have become aware of the consequences of their actions and how these actions might affect the world in the future. They might have come to the realization they must do more in order to avoid these consequences. The effect of conversation, therefore, might not have been limited to an increase in empathy, but also to dispassionate reasoning [102].

#### Age differences

Conversation seems to have differing effects on altruistic behavior across age groups. In round 1 and round 2, the old age group donated more money in the Dictator Game compared to the young age group. We believe the reason for this difference is due to financial stability.

In an open-ended questionnaire item: *Why did you decide on this split in the Dictator Game?*, some of the participants in the young age group mentioned that the reason they didn’t share any of the endowment (or a small amount) is due to financial reasons. Some of their answers were: “Because I cannot afford to let go of any part of my endowment.”, “Because I don’t have that much money to spare, and even if I wanted to donate, it would not be easy for me.”, “Because I would not normally donate to any cause, considering my financial situation. And I thought that I should not be swept away even if we had a face-to-face conversation.”

Such answers show that for students, sharing a part of their endowment is difficult regardless of how convinced they were with the scenario. Answers citing financial reasons were only present in the young age group and not in the old age group, which might be a reason why older adults were more willing to share their endowment even when the belief level between the old age group and the young age group was not significantly different in round 2.

#### Media differences

As a result of the analysis of the Dictator Game results, we were only able to see a significant difference between the amounts shared with the speaker and human for only the old age group in round 2. Older adults seemed to give more money in round 2 to the speaker compared to the human. However, overall, the effect of appearance on Dictator Game giving seems to be negligible.

We can see an effect of appearance from the open-ended questionnaire item: *Why did you decide on this split in the Dictator Game?*, two participants claimed that the reason they gave money in the human condition was that they thought the human medium was attractive. This shows that the results in the Dictator Game can be biased due to the appearance of the opponent in the game. Additionally, some participants claimed that the reason they gave money was because they felt pressured to give money due to the presence of the medium in front of them. These responses only appeared in the human and robot condition, but not in the speaker condition. These answers reveal that it might be quite complicated to isolate the effect of appearance on altruistic behavior simply through Dictator Game giving.

The results indicate that conversation might be effective at increasing altruistic behavior regardless of the medium used. As robots appear to be just as effective as humans at eliciting altruistic responses in humans, this can significantly affect the role social robots can play in society. In addition to mitigating the bias that human appearance might elicit from participants. Differences between the robot and speaker might become apparent as the conversation becomes more developed to elicit more empathy and believability from participants, where anthropomorphization might become the decisive factor separating these two conditions.

### Reinforcing altruistic behavior

#### Empathy

Empathy, as an innate trait, is adjustable and can be strengthened with strategic educational approaches. Strengthening empathy might be an effective strategy, as it appears to be a good moderator of the effect of conversation on altruistic behavior. According to Jones [103], successful learners need to be empathetic in addition to being knowledgeable, self-determined, and strategic. Empathy training has been shown to be successful in enhancing both cognitive and affective empathy in both children and adults, in addition to leading to more prosocial behavior [104]. Bratitsis et al. [105] developed an educational robot for empathy training with children as their target group. They discussed the use of their robot to increase empathy toward children with ASD and to decrease bullying. The use of such social robots in classrooms can be very beneficial in reinforcing empathy from a young age. This in turn can lead to more understanding and awareness of environmental issues as they become more and more informed on such matters.

Even though empathy, whether innate or induced, seems to be effective in reinforcing altruistic behavior, it is important to mention that empathy is limited in this capacity [102, 106]. Reinforcement of altruistic behavior would benefit from a rational system of thought and decision making over blindly following emotional intuitions.

#### Generational differences

**Fig 10:**
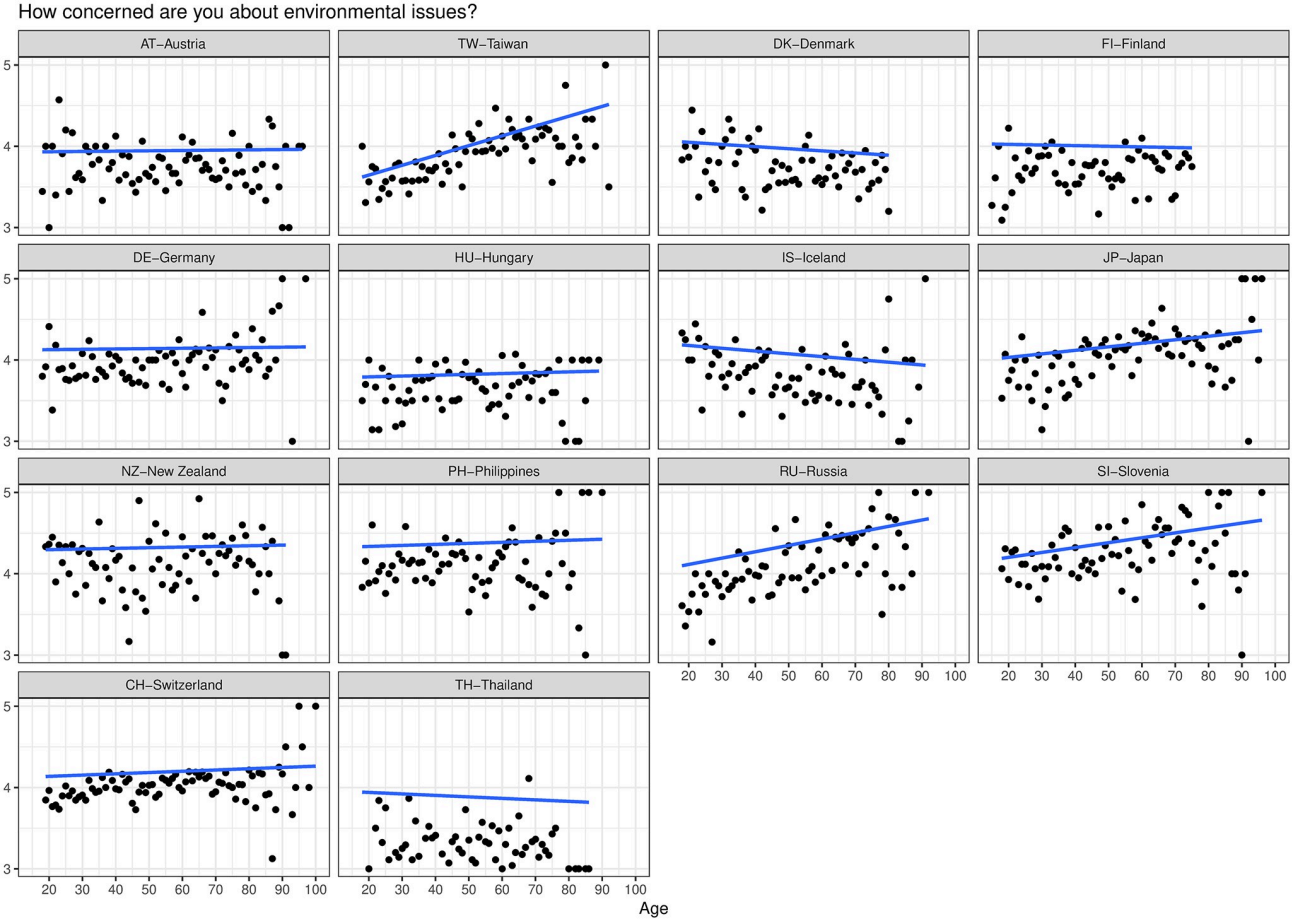
Environmental concern as a function of age. Environmental concern as a function of age in different countries using data published by the 2020 Environment survey by the International Social Survey Program [109]. In Japan, environmental concern seems to rise with age.

Different approaches might be more suitable for different age groups. Interstingly, our data showed that young people in Japan seem to have less interest and awareness of environmental issues compared to older adults. This can be seen in previous studies on the Japanese population [107, 108]. From the 2020 Environment survey by the International Social Survey Program (ISSP) [109], the data also show higher environmental concern in Japan as age increases as shown in Fig 10. A possible explanation is that older adults in Japan today have lived through periods of history (the 1950s-1960s) where there were many civil movements as a result of pollution, and the spread of pollution-related diseases (mercury and cadmium poisoning) in addition to anti-nuclear protests relating to the nuclear tests at Bikini Atoll and its effects on fishing in Japan [110]. Older adults today might have taken part in these movements or just having lived through those periods resulted in them having a higher environmental concern than young people today. Comparatively, there seems to be a lack of education on environmental issues at a young age in Japan. However, our results show that this can improve through dialogue.

Older adults showed more environmental concern and awareness compared to younger people. Their relatively high donations show that they want to contribute to environmental protection. Apart from donations, older adults want to participate and contribute to society through volunteer work [111]. In that study, older adults had conversations with children by teleoperating a robot. This might be a good role for older adults where they can educate younger people at an early age. They can discuss topics related to environmental issues and protection with kids by teleoperating a robot placed at schools. This could be very beneficial for kids, as this raises awareness at a young age, and it is a good role for older adults who can contribute to creating an environmentally friendly community regardless of their physical abilities.

#### Collectivism

The scores of the questionnaire item 21, “Based on what you know about the risks related to environmental pollution, is there a possibility that you may recommend your friends or colleagues to reduce global warming?”, seem awfully low. There seems to be a hesitation to talk about these issues. This might be attributed to the collectivistic nature of the Japanese society. Young and old people are willing to learn and change their behavior, but they might not be willing to lead or educate others. Social robots, in this case, can take the initiative and have conversations with people about environmental issues. Additionally, people can be connected to each other through robots to have discussions together. People can control these robots and have conversations with a layer of anonymity [111], which might make them more comfortable knowing that their identity is concealed.

## Limitations

One of the biggest limitations of this study is the sample size. Sixty participants were recruited for this study is fare from idead to make any conclusive predictions on the effect of conversation. Another limitation of this study is the homogeneity of participants who were all Japanese and fell within two age groups only. Finally, the likeability of the appearances of the three media was not tested in this study, and will be included in our future studies.

## Conclusion and future work

In this study, we analyzed whether conversation is likely to encourage altruistic conduct toward future generations and identify the questions that need to be answered in future research. Our main research question was stated as follows: *“Does dialogue influence altruistic behavior toward future generations?”*. The findings suggest that dialogue seems to positively influence altruistic behavior toward future generations. Our sub-question was: *“If conversation is an influencing factor, are there differences in its effects based on age, appearance, and personality?”*. The results show that the effect of conversation on altruistic behavior was different across age groups but not across different media, and personality factors showed no effect on altruistic behavior except for affective empathy which showed to positively moderate the effect of conversation on altruistic behavior.

For our future work, we wish to continue investigating the effect of appearance on altruistic behavior with a bigger number of participants, in addition to the long-term effects of conversation on altruistic behavior. We would also like to investigate cultural effects on altruistic behavior, or how these results might vary if the experiment was conducted in different countries. Finally, we will also investigate a fundamental issue of whether there are any differences between altruism toward the present and future generations.

## Supporting information

S1 File The script used in the interactive conversation for round 2. (PDF)

S2 File The full list of questionnaire items included in all the questionnaires in this study. (PDF)

S3 File The complete tables of all the results of the data analysis. (PDF)

S1 Dataset The raw data used for data analysis for replication purposes. (XLSX)
