# Supplementary material for: The effect of conversation on altruism: A comparative study with different media and generations
Source: PLoS One. 2024 Jun 14;19(6):e0301769. doi: 10.1371/journal.pone.0301769 (PMC11178171; doi:10.1371/journal.pone.0301769)
Supplement: S1 File — (PDF) [file pone.0301769.s001.pdf]

Conversation Script

The following is the script for the conversation between the participant and the medium. The conversation was originally written and conducted in Japanese. A translation is provided here.

- **Medium:** Hello. What is your name?
  Wait for participant's response
- **Medium:** Nice to meet you. My name is Mirai. What year do you live in now?
  Wait for participant's response
- **Medium:** Oh, I see. I live in the year 2220. What is life like in 2022? Please tell us what you like about living in 2022.
  Wait for participant's response
- **Medium:** That's interesting. There are some very nice things about 2220. We have flying cars that make transporting things so much easier and more convenient. As oil is depleted and disappearing, we are using more efficient energy sources. Some countries are building and operating their own mini-suns, small suns, as energy sources. In your time, are there any countries that are working on prototypes of small artificial suns?
  Wait for participant's response
- **Medium:** Nowadays, windmills are also much more efficient. There are vast wind farms on the ocean that can power entire cities. We have space elevators that can take us to the moon in 15 minutes. Well, it would be quite expensive to actually use it. Do you have hope for the lives of future generations?
  Wait for participant's response
- **Medium:** I see. What's the one thing you don't like about your life in 2022?
  Wait for participant's response
- **Medium:** Well, that's too bad. In 2220, the earth is very hot and it is very difficult to find food. Our lives have been severely affected by violent climate change for the past 100 years. In many places around the world, the temperature is getting hotter and the air feels heavier. It is very difficult to leave the building, walk around, breathe fresh air, etc. You start coughing and can't stop. Do you have any equipment that you wear on your body whenever you go outside your home?
  Wait for participant's response
- **Medium:** Is that so. It is getting hotter and hotter all over the world. Controlling the environment is beyond our control. We have passed the tipping point. Even those living in relatively mild climates, such as Canada and Scandinavia, are severely vulnerable. Severe tornadoes, flash floods, mudslides, and wind storms are a daily occurrence. Is it hard to find food in your time?
  Wait for participant's response
- **Medium:** Oh, I see. Is the situation the same everywhere in the world?
  Wait for participant's response
- **Medium:** Well, in 2220, food shortages are a big problem everywhere in our lives. Especially in the last 100 years, food production has changed dramatically from month to month, season to season, depending on where you live. Humans have continued to emit large amounts of carbon dioxide into the atmosphere. This has affected the oceans. Carbon dioxide dissolves in seawater and acts as an acid. As the water became more acidic, it destroyed the ecosystems of marine organisms. As a result, all countries banned fishing because of the impact on humans. In your time, can you still eat fish?
  Wait for participant's response

- **Medium:** Oh, is that so. Do you want to think about how our current carbon emissions will affect future ecosystems and food?
  Wait for participant's response
- **Medium:** Well, in 2220, more people will be hungry than ever before. The tyranny of food supply and demand has been relentlessly suppressed. Food became scarce and expensive as prices skyrocketed and food became a luxury for the rich. Most people stole food to survive. Today, however, food is almost impossible to come by. If you had to deal with these food shortages, how would you deal with them?
  Wait for participant's response
- **Medium:** For us, food shortages are a matter of survival. We have to steal whatever we can find. If we can find insects, we will eat whatever we can find. Some of us live on cannibalism as a means of survival. I remember when I lived in 2022, not many people believed in climate change. What do you think about the fact that not many people really believed in climate change in your time?
  Wait for participant's response
- **Medium:** There is a lot of talk now about the end of the human species. For many people, there is only one thing that is uncertain: when will they survive. How long will they survive, and how many more generations of humans will see the light of day. In your time, have these things been discussed yet?
  Wait for participant's response
- **Medium:** Mankind, their suicide, is the most obvious indication of the intense despair that is now spreading around. But there is also a bottomless sense of loss, unbearable guilt, and intense resentment toward previous generations that did not do what was necessary to avoid this unstoppable disaster. 2220 years later, we are in this situation. It has been a pleasure speaking with you today, people from the past. It was also nice to hear from a future generation, as it was for you. Goodbye.
