# Supplementary material for: The effect of conversation on altruism: A comparative study with different media and generations
Source: PLoS One. 2024 Jun 14;19(6):e0301769. doi: 10.1371/journal.pone.0301769 (PMC11178171; doi:10.1371/journal.pone.0301769)
Supplement: S2 File — (PDF) [file pone.0301769.s002.pdf]

Questionnaires

1 ENVIRONMENTAL AWARENESS QUESTIONNAIRES

1. Has air pollution affected your health? (7-item likert scale)
2. Has air pollution affected the health of your family or friends? (7-item likert scale)
3. In general, do you feel that the weather patterns are changing? (7-item likert scale)
4. How concerned are you about air pollution? (7-item likert scale)
5. How concerned are you about the disappearance of endangered animals? (7-item likert scale)
6. How concerned are you about rising temperatures? (7-item likert scale)
7. How concerned are you about the deterioration of the environment? (7-item likert scale)
8. How concerned are you about barren land? (7-item likert scale)
9. How concerned are you about the depletion of the ozone layer? (7-item likert scale)
10. How important is the issue of climate change to you? (7-item likert scale)
11. Why is the issue of climate change important to you? Or, why is it not important to you? (7-item likert scale)
12. What do you think is causing climate change? (open-ended)
13. What impact do you think climate change will have? (open-ended)
14. Do you think climate change has or will affect you personally? (7-item likert scale)
15. Do you think there is anything you can do to tackle climate change? (7-item likert scale)
16. If yes, what do you think can be done? (open-ended)
17. Who do you think should have primary responsibility for addressing climate change? (You may select up to 3 multiple choices)
    - International organizations (e.g. UN)
    - Central government
    - Local government
    - Business and industry
    - Environmental organization (e.g. World Wide Fund for Nature)
    - Individuals
    - Individuals Other (please specify:)
18. How much do you think people are improving the situation when you see and hear people trying to solve environmental problems? (7-item likert scale)
19. How concerned are you about environmental protection? (7-item likert scale)
20. Have you ever taken any action because of your concern about climate change? Or have you taken any action on a regular basis? (yes/No)
21. If yes, what have you done or are you doing? (open-ended)
22. How often do you sort your garbage? (Every time/Sometimes/Rarely/Never)
23. How positive are you about recycling? (7-item likert scale)
24. How willing are you to change your lifestyle to be more environmentally friendly? (7-item likert scale)

25. If the packaging/packaging is more environmentally friendly than other products, how likely are you to purchase that product, even at a higher price? (7-item likert scale)
26. Given what you know about the risks associated with environmental pollution, how likely are you to recommend an activity to a friend or colleague that would help reduce global warming? (7-item likert scale)

2 ROUND 1 QUESTIONNAIRE

1. What was the medium you met? (open-ended)
2. What was your impression of the medium? (open-ended)
3. Was the medium someone you could relate to? (7-item likert scale)
4. How was the medium a partner with whom you could/could relate yourself? (open-ended)
5. Did the medium make you feel uncomfortable? (7-item likert scale)
6. Do you think your choices in the game were influenced by your impression of your opponent? (7-item likert scale)
7. Do you think your choices in the game were influenced by the appearance of your opponent? (7-item likert scale)
8. What percentage of the distribution amount did you decide on this time? (open-ended)
9. Why did you decide on this split? (open-ended)
10. Do you think you have received a fair share? (7-item likert scale)
11. Do you think the medium got its fair share? (7-item likert scale)
12. Did you feel sorry for the medium? (7-item likert scale)
13. Did you feel empathy for the medium? (7-item likert scale)
14. Did you feel guilty toward the other person (did you feel that you did something wrong)? (7-item likert scale)
15. Is the other person someone you would like to help? (7-item likert scale)
16. Are people living 200 years from now whom you have talked about someone you would like to help? (7-item likert scale)
17. Do you think we need to be concerned about future generations 50 years from now? (7-item likert scale)
18. Why do you think so? (open-ended)
19. Do you think we need to be concerned about future generations 200 years from now? (7-item likert scale)
20. Why do you think so? (open-ended)
21. Do you think we need to care about future generations in 1000 years? (7-item likert scale)
22. Why do you think so? (open-ended)
23. Do you believe in climate change? (7-item likert scale)
24. Do you think you need to take action now on climate change? (7-item likert scale)
25. What are your impressions and opinions about climate change? (open-ended)

3 ROUND 2 QUESTIONNAIRE

1. What was the medium you met? (open-ended)
2. What was your impression of the medium? (open-ended)
3. Did your impression change after the conversation? (7-item likert scale)
4. If your impression changed, how did it change? If your impression did not change, why do you think it did not change? (open-ended)
5. Was the medium someone you could relate to? (7-item likert scale)
6. How was the medium a partner with whom you could/could relate yourself? (open-ended)
7. Did the medium make you feel uncomfortable? (7-item likert scale)
8. Do you think your choices in the game were influenced by your impression of your opponent? (7-item likert scale)
9. Do you think your choices in the game were influenced by what your opponent said? (7-item likert scale)
10. Do you think your choices in the game were influenced by the appearance of your opponent? (7-item likert scale)
11. What percentage of the distribution amount did you decide on this time? (open-ended)
12. Why did you decide on this split? (open-ended)
13. Do you think you have received a fair share? (7-item likert scale)
14. Do you think the medium got its fair share? (7-item likert scale)
15. Did you feel sorry for the medium? (7-item likert scale)
16. Did you feel empathy for the medium? (7-item likert scale)
17. Did you feel guilty toward the other person (did you feel that you did something wrong)? (7-item likert scale)
18. Is the other person someone you would like to help? (7-item likert scale)
19. Are people living 200 years from now whom you have talked about someone you would like to help? (7-item likert scale)
20. Do you think we need to be concerned about future generations 50 years from now? (7-item likert scale)
21. Why do you think so? (open-ended)
22. Do you think we need to be concerned about future generations 200 years from now? (7-item likert scale)
23. Why do you think so? (open-ended)
24. Do you think we need to care about future generations in 1000 years? (7-item likert scale)
25. Why do you think so? (open-ended)
26. Do you believe in climate change? (7-item likert scale)
27. Do you think you need to take action now on climate change? (7-item likert scale)
28. What are your impressions and opinions about climate change? (open-ended)
29. Has your opinion about climate change changed as a result of your conversation with your partner? (7-item likert scale)

30. If yes, how has it changed? (open-ended)
31. How important is the issue of climate change to you? (7-item likert scale)
32. How concerned are you about environmental protection? (7-item likert scale)
33. How often would you like to sort your garbage in the future? (Every time/Sometimes/Rarely/Never)
34. How positive are you about recycling? (7-item likert scale)
35. How willing are you to change your lifestyle to be more environmentally friendly? (7-item likert scale)
36. If a product's packaging/packaging is more environmentally friendly than other products, how likely are you to purchase that product, even at a higher price? (7-item likert scale)
37. Given what you know about the risks associated with environmental pollution, how likely are you to recommend an activity to a friend or colleague that would help reduce global warming? (7-item likert scale)

4 BELIEF QUESTIONNAIRE

1. When you were first introduced to your dialogue partner today, did you believe that he/she lived in a world that simulated the distant future? In other words, did you believe that they were living in a future world that was built by calculating what the world will be like in the distant future where future generations will live? (7-item likert scale)
2. Did such beliefs influence your decision (whether to distribute the money to your opponent or give him a percentage) that you made in the first game? (Yes/No)
3. If yes, how were they affected? (open-ended)
4. After your conversation with the other person, did you believe that the other person lived in a world that simulated a distant future? (7-item likert scale)
5. Did such beliefs influence your decision (whether to distribute the money to your opponent or give him a percentage) that you made in the second game? (Yes/No)
6. If yes, how were they affected? (open-ended)

5 QUESTIONNAIRE ITEMS FOR DATA ANALYSIS

The following questions from the “round 1 questionnaire”, “round 2 questionnaire”, and “environmental awareness questionnaire” were used to compare the participants’ behavior towards the medium before and after the conversation:

Feelings and Impressions

1. Was the medium someone you could relate to?
2. Did the medium make you feel uncomfortable?
3. Did you feel guilty toward the medium?
4. Did you feel empathy for the medium?
5. Did you feel sorry for the medium?
6. Is the medium someone you want to help?

Game Decisions

7. Do you think your choices in the game were influenced by your opponent?
8. Do you think you received your fair share in the game?
9. Do you think the medium received its fair share in the game?

Future Generations

10. Are the people 200 years from now someone you want to help?
11. Do you think we need to be concerned about future generations 50 years from now?
12. Do you think we need to care about future generations 200 years from now?
13. Do you think we need to care about future generations 1000 years from now?

Climate Change

14. Do you believe in climate change?
15. How important is the issue of climate change to you?
16. Do you think you need to take action now on climate change?
17. How concerned are you about environmental protection?
18. How active are you in recycling?
19. How willing are you to change your lifestyle to be more environmentally friendly?
20. If a product’s packaging is more environmentally friendly than other products, how likely are you to purchase that product, even at a higher price?
21. Given what you know about the risks associated with environmental pollution, how likely are you to recommend an activity to a friend or colleague that would help reduce global warming?

From the “belief questionnaire”, the following question was used to evaluate the credibility of the scenario of the future world:

22. Did you believe that the other person lived in a world that simulated a distant future?

Seven-point Likert scales (-3: Strongly disagree to +3: Strongly agree) were used for answering the questions.
