# Supplementary material for: The effect of conversation on altruism: A comparative study with different media and generations
Source: PLoS One. 2024 Jun 14;19(6):e0301769. doi: 10.1371/journal.pone.0301769 (PMC11178171; doi:10.1371/journal.pone.0301769)
Supplement: S3 File — (PDF) [file pone.0301769.s003.pdf]

Data Analysis

1 QUESTIONNAIRES

Table S1. Results of the Questionnaires. The data was analyzed with the Wilcoxon signed-rank two-tailed test for comparing ranks of paired data. The question numbers refer to the questions in the "Questionnaire Items for Data Analysis" section of the "Questionnaires" supplementary material.

Question number	Round 1		Round 2		95% Conf.Int. (lower)	95% Conf.Int. (upper)	p-value (two-tailed)	Effect size (r)
	Mdn	IQR	Mdn	IQR				
**Feelings and Impressions**								
1	-1.0	2.25	0.0	2.00	-2.00	-0.50	$p < 0.01$	0.34
2	-1.0	3.00	-2.0	3.00	0.00	1.50	$p = 0.04$	0.26
3	-2.0	3.00	0.0	2.00	-2.00	-1.00	$p < 0.0001$	0.53
4	0.0	2.00	1.0	2.00	-2.00	-1.00	$p < 0.0001$	0.57
5	-1.0	3.00	0.5	3.00	-3.00	-1.50	$p < 0.0001$	0.54
6	0.0	2.00	1.0	1.00	-2.50	-1.00	$p < 0.0001$	0.60
**Game Decisions**								
7	0.0	3.00	1.0	3.25	-2.00	0.00	$p = 0.02$	0.29
8	2.0	3.00	1.50	3.00	-1.50	1.00	$p = 0.86$	0.06
9	0.5	2.00	0.0	2.00	-1.00	1.50	$p = 0.56$	0.01
**Future Generation**								
10	1.0	2.00	1.0	1.00	-1.50	-1.00	$p < 0.001$	0.49
11	2.5	1.00	2.0	1.00	-0.50	0.50	$p = 0.91$	0.02
12	1.0	3.00	2.0	2.00	-2.00	0.00	$p < 0.01$	0.36
13	0.5	2.00	1.0	2.00	-1.50	-0.50	$p < 0.01$	0.42
**Climate Change**								
14	2.0	2.00	2.0	2.00	0.00	1.00	$p = 0.55$	0.06
15	2.0	1.00	2.0	1.00	0.00	0.50	$p = 0.47$	0.09
16	1.0	1.00	1.0	1.00	-1.00	0.00	$p = 0.09$	0.30
17	1.0	1.00	1.5	1.00	-1.50	-1.00	$p < 0.0001$	0.34
18	1.0	1.00	2.0	2.00	-1.00	-1.00	$p < 0.001$	0.47
19	1.0	1.00	1.0	1.00	-1.00	0.00	$p < 0.01$	0.37
20	0.0	2.00	1.0	2.25	-1.50	0.00	$p < 0.01$	0.35
21	0.0	2.00	1.0	1.25	-1.50	-1.00	$p < 0.0001$	0.55

2 PERSONALITY

Table S2. Results of the linear mixed effects model, where the amount percentage in the Dictator Game is the dependent variable, and the personality factors of the NEO-FFI test are the independent variables. The table shows regression coefficients (B), standard errors, t statistics, and p values.

NEO-FFI				
**Fixed effects**	**B**	**Std. Error**	**t**	**p-value**
Extraversion				
Intercept	27.30	29.25	0.93	$p = 0.35$
Time	6.94	24.94	0.28	$p = 0.78$
Extraversion	0.17	0.86	0.20	$p = 0.84$
Time:Extraversion	0.13	0.73	0.18	$p = 0.86$
Neuroticism				
Intercept	5.68	28.33	0.20	$p = 0.84$
Time	52.37	23.65	2.21	$p = 0.03$
Neuroticism	0.74	0.75	0.98	$p = 0.33$
Time:Neuroticism	-1.10	0.63	-1.75	$p = 0.09$
Openness to Experience				
Intercept	75.96	38.87	1.95	$p = 0.06$
Time	-11.35	33.21	-0.34	$p = 0.73$
Openness	-1.12	1.02	-1.10	$p = 0.27$
Time:Openness	0.60	0.87	0.69	$p = 0.50$
Agreeableness				
Intercept	4.34	34.95	0.12	$p = 0.90$
Time	-0.11	30.01	-0.004	$p = 0.99$
Agreeableness	0.73	0.88	0.83	$p = 0.41$
Time:Agreeableness	0.29	0.76	0.38	$p = 0.70$
Conscientiousness				
Intercept	38.28	26.38	1.45	$p = 0.15$
Time	2.60	22.46	0.12	$p = 0.91$
Conscientiousness	-0.15	0.79	-0.20	$p = 0.85$
Time:Conscientiousness	0.26	0.67	0.39	$p = 0.70$

Table S3. Results of the linear mixed effects model, where the amount percentage in the Dictator Game is the dependent variable, and the personality factors of the HEXACO-PI-R test are the independent variables. The table shows regression coefficients (B), standard errors, t statistics, and p values.

**HEXACO-PI-R**				
**Fixed effects**	**B**	**Std. Error**	**t**	**p-value**
Honesty-Humility				
Intercept	9.24	18.05	0.51	$p = 0.61$
Time	-14.97	15.64	-0.96	$p = 0.34$
Honesty-Humility	0.66	0.49	1.35	$p = 0.18$
Time:Honesty-Humility	0.73	0.43	1.71	$p = 0.09$
Emotionality				
Intercept	47.03	23.50	2.00	$p = 0.05$
Time	23.48	20.10	1.17	$p = 0.25$
Emotionality	-0.41	0.69	-0.60	$p = 0.55$
Time:Emotionality	-0.36	0.59	-0.61	$p = 0.54$
Extraversion				
Intercept	21.21	17.12	1.24	$p = 0.22$
Time	16.87	14.61	1.15	$p = 0.25$
Extraversion	0.40	0.56	0.71	$p = 0.48$
Time:Extraversion	-0.18	0.48	-0.38	$p = 0.70$
Agreeableness				
Intercept	27.10	19.98	1.36	$p = 0.18$
Time	28.54	16.90	1.69	$p = 0.10$
Agreeableness	0.18	0.60	0.31	$p = 0.76$
Time:Agreeableness	-0.52	0.51	-1.03	$p = 0.31$
Conscientiousness				
Intercept	15.07	21.46	0.70	$p = 0.49$
Time	8.21	18.43	0.45	$p = 0.66$
Conscientiousness	0.55	0.65	0.85	$p = 0.40$
Time:Conscientiousness	0.10	0.56	0.17	$p = 0.86$
Openness to Experience				
Intercept	23.25	16.19	1.44	$p = 0.16$
Time	18.03	13.80	1.31	$p = 0.20$
Openness	0.29	0.47	0.63	$p = 0.53$
Time:Openness	-0.20	0.40	-0.50	$p = 0.62$

Table S4. Results of the linear mixed effects model, where the amount percentage in the Dictator Game is the dependent variable, and the empathy factors of the MES test are the independent variables. The table shows regression coefficients (B), standard errors, t statistics, and p values.

MES				
**Fixed effects**	**B**	**Std. Error**	**t**	**p-value**
Affective				
Intercept	72.67	33.09	2.20	$p = 0.03$
Time	-71.25	26.43	-2.70	$p = 0.009$
Affective	-0.89	0.74	-1.20	$p = 0.24$
Time:Affective	1.86	0.59	3.14	$p = 0.003$
Cognitive				
Intercept	63.99	26.42	2.42	$p = 0.02$
Time	37.79	22.84	1.65	$p = 0.10$
Cognitive	-0.91	0.77	-1.18	$p = 0.24$
Time:Cognitive	-0.78	0.67	-1.17	$p = 0.25$
